# Enamel Matrix Derivative Inhibits Adipocyte Differentiation of 3T3-L1 Cells via Activation of TGF-βRI Kinase Activity

**DOI:** 10.1371/journal.pone.0071046

**Published:** 2013-08-12

**Authors:** Reinhard Gruber, Dieter D. Bosshardt, Richard J. Miron, Anja C. Gemperli, Daniel Buser, Anton Sculean

**Affiliations:** 1 Department of Periodontology, School of Dental Medicine, University of Bern, Bern, Switzerland; 2 Department of Oral Surgery and Stomatology, School of Dental Medicine, University of Bern, Bern, Switzerland; 3 Laboratory of Oral Cell Biology, School of Dental Medicine, University of Bern, Bern, Switzerland; 4 Robert K. Schenk Laboratory of Oral Histology, School of Dental Medicine, University of Bern, Bern, Switzerland; 5 Institut Straumann, Basel, Switzerland; Baylor College of Medicine, United States of America

## Abstract

Enamel matrix derivative (EMD), an extract of fetal porcine enamel, and TGF-β can both suppress adipogenic differentiation. However, there have been no studies that functionally link the role of EMD and TGF-β in vitro. Herein, we examined whether TGF-β signaling contributes to EMD-induced suppression of adipogenic differentiation. Adipogenesis was studied with 3T3-L1 preadipocytes in the presence of SB431542, an inhibitor of TGF-βRI kinase activity. SB431542 reversed the inhibitory effect of EMD on adipogenic differentiation, based on Oil Red O staining and mRNA expression of lipid regulated genes. SB431542 also reduced EMD-stimulated expression of connective tissue growth factor (CTGF), an autocrine inhibitor of adipogenic differentiation. Moreover, short interfering (si)RNAs for CTGF partially reversed the EMD-induced suppression of lipid regulated genes. We conclude that the TGF-βRI - CTGF axis is involved in the anti-adipogenic effects of EMD in vitro.

## Introduction

Emdogain® is the commercial name for the combination of enamel matrix derivatives (EMD) isolated from the tooth germs of 6-month old piglets and the vehicle propylene glycol alginate (PGA) (Institut Straumann, Basel, Switzerland, formerly Biora, Malmö, Sweden). Emdogain® is approved to support periodontal tissue regeneration [1]. Histological and clinical data have indicated that the use of Emdogain® in combination with palatal subepithelial connective tissue grafts (CTG) may enhance periodontal wound healing/regeneration and to additionally improve the clinical outcomes when compared to the use of CTG alone [2]–[4]. Periodontal tissues and connective tissue grafts both contain mesenchymal cells that can become adipocytes [5]–[7]. However, adipogenic differentiation is unwanted when a regain of periodontal structures or the formation of a collagen-rich matrix is desired, respectively. A first clue that Emdogain® can suppress adipogenic differentiation comes from in vitro studies with the mouse multipotent myoblast cell line C2C12 [8] and periodontal ligament fibroblasts [6]. The underlying cellular mechanisms however are poorly defined [9], [10].

Transforming growth factor-beta1 (TGF-β) signaling is among the key mechanisms that can mediate at least part of the in vitro cellular responses to EMD and Emdogain® [11]–[14]. Recombinant TGF-β inhibits adipocyte differentiation as exemplified by the suppression of lipid droplets and the expression of adipogenic genes such as peroxisome proliferator-activated receptor γ (PPARγ), fatty acid binding protein 4 (FABP4), thrombospondin receptor (CD36), and leukotriene C4 synthase (LTC4s) in the pre-adipogenic 3T3-L1 clonal cell line [15], [16]. TGF-β binding to type I and type II receptor kinases (TGF-βR) activates Smad2 and Smad3 signaling [17]. TGF-βR can also signal through mitogen-activated protein kinases, including ERK, c-Jun N-terminal kinase (JNK) and p38, as well the PI3K pathway [18]. Smad [19] and mitogen-activated protein kinase [20] signaling are involved in TGF-β -mediated inhibition of adipogenesis. Also EMD can activate signaling via Smad2 and JNK [21]. Together, these data led to the hypothesis that the suppression of adipogenic differentiation by EMD may involve TGF-β signaling.

Consistent with this hypothesis is that both, TGF-β and Emdogain® increase the expression of connective tissue growth factor (CTGF) also known as CCN2 [14], [22], [23]. CTGF inhibits adipocyte differentiation [23] and CTGF can mediate the cellular responses to TGF-β, including the inhibition of adipocyte differentiation [16]. Moreover, enamel matrix derivative can also increase CTGF expression via TGF-β activity in osteoblastic cells [14]. SB431542, a TGF-β receptor antagonist and a JNK antagonist can inhibit CTGF expression induced by TGF-β1 in fibroblasts [24], [25]. It is thus reasonable to hypothesize that the expected suppression of adipogenic differentiation by EMD requires TGF-β signaling and involves CTGF expression. Therefore, the aim of this study was to test this hypothesis by means of the pre-adipogenic 3T3-L1 cell line.

## Materials and Methods

### Adipogenic Differentiation

The 3T3-L1 murine preadipocyte cell line was kindly donated by Christian Wolfrum ([26]; ETH Zürich, Switzerland) and cultured in a humidified atmosphere at 37°C in growth medium consisting of DMEM (Invitrogen Corporation, Carlsbad, CA, USA), 10% fetal calf serum (FCS; Invitrogen) and antibiotics (Invitrogen). Mouse subcutaneous adipose tissue was obtained from the inguinal region and cells were isolated by 0.1% collagenase I (Sigma) digestion. Cells were plated in growth medium at 30,000 cells/cm2 into culture dishes. The following day, cells were incubated in growth medium containing 0.5 mM 1-methyl-3-isobutyl-xanthine (Sigma), 1 µM dexamethasone (Sigma) and 1 µg/ml insulin (Calbiochem, Merck Millipore; MA). To further stimulate adipogenesis, 10 µM indomethacin (Sigma) and 10 µM rosiglitazone (Sigma) were added to the growth medium [27]. If not otherwise indicated, cells were cultivated for 5 days.

### Test Compounds

Cells were incubated with Emdogain® at dilutions equivalent to 100 mg EMD/ml or the respective carrier propylene glycol alginate (PGA; kindly provided by Dr. Graf; Institut Straumann AG, Basel, Switzerland). Emdogain® containing 30 mg enamel matrix derivative (EMD)/ml PGA (approximately 6,5% wt. PGA, pH 3.7) and the respective vehicle were dissolved in serum-free medium to 10 mg EMD/ml and kept a 4°C for further dilution. For indicated experiments, Emdogain® (10 mg/ml) was heat treated at 96°C for 3 min [28]. 3T3-L1 cells were also exposed to Emdogain® and TGF-β for 24 hours before further cultivation in adipogenic medium. Recombinant human (rh) TGF-β1 was purchased from Prospec (Ness-Ziona, Israel). SB431542 (TGF-β receptor antagonist; IC50 = 94 nM) and SB600125 (JNK inhibitor; IC50 = 40–90 nM) were purchased from Santa Cruz Biotechnology, SCBT; Santa Cruz, CA).

### TGF-β1 Enzyme Immunoassay

The immunoassay for the determination of TGF-β1 was obtained from Enzo Life Sciences AG (Lausen, Switzerland). Emdogain was diluted to give 1 mg EMD/ml and processed to obtain a bioactive form that can be detected by the assay. In brief, twenty µL of 1N HCl were added to 100 µL EMD, and after ten minutes neutralized with 20 µL 1.2 N NaOH/0.5 M HEPES. These samples were subjected to immunoassay and TGF-β1 concentration was calculated based on a calibration curve.

### Oil Red O Staining

Cells were fixed with 10% neutral buffered formalin, washed with 60% isopropanol, and stained with Oil Red O (0.5%; Sigma). Cells were rinsed several times with tap water and subjected to microscopic analysis and were photographed.

### Gene Expression Analysis

Cellular RNA was isolated using an RNAqueous-Micro Kit containing DNAse I (Ambion, Life Technologies). RNA was quantified (Nanodrop 2000c; Thermo Scientific, Waltham, MA, USA). Reverse transcription (RT) was performed with a high-capacity cDNA RT-kit (Applied Biosystems, Foster City, CA) and PCR was done with TaqMan® universal PCR Master Mix (Applied Biosystems) on a 7500 Real-Time PCR System (Applied Biosystems). For screening, the TaqMan® Array Mouse Lipid Regulated Genes 96-well Plate (Applied Biosystems), a panel of assays for genes controlling sterol metabolism, fatty acid metabolism, lipid droplet, and transcription factors was used. Further probes were obtained from the TaqMan® Gene Expression Assays service (Applied Biosystems, Mm01184322_m1 PPARγ; Mm00521864_m1 Ltc4s; Mm00445878_m1 Fabp4; Mm00432403_m1 Cd36; Mm01192932_g1 CTGF; Mm01250458_m1 Alox15). For the expression of TGF-β, we used designed primers (forward tggagcaacatgtggaactc; reverse gtcagcagccggttacca) and SYBR Green as detection signal. The mRNA levels were calculated by normalizing to the housekeeping gene beta actin using the ΔCt method.

### Western Blot Analysis

For phospho-Smad3 staining, 3T3-L1 cells were washed with phosphate buffered saline, serum-starved for over night and then treated with Emdogain® and TGF-β for 1 hours. For PPARγ staining, 3T3-L1 cells were grown in adipogenic medium with and without Emdogain® for three days. Cells were lysed in SDS-buffer containing protease inhibitors. Cell extracts were separated by SDS-PAGE and transferred onto nitrocellulose membranes. Membranes were blocked in a supplied buffer (LI-COR Biosciences; Lincoln, NE). Binding of the antibody raised against phospho-Smad3 (Ser423/425) (Cell Signaling Technology, Danvers, MA), PPARγ (E-8) and β-actin (C-4) (both SCBT) were detected with the appropriate secondary antibody directly labeled with near-infrared dyes and detected with the appropriate imaging system (LI-COR Biosciences; Lincoln, NE).

### Transfection with siRNA

CTGF siRNA, mock siRNA and the transfection agent were purchased from SCBT. The transfection protocol was followed according to the instructions of the manufacturer. Inhibition efficacy was determined by Western blot analysis for CTGF. Transfected cells were exposed to Emdogain® at 100 ng/ml and TGF-β at 10 ng/ml in serum-free medium for 24 hours. Gene expression analysis was performed targeting PPARγ. Transfected cells were also tested for their potential to provoke Oil Red O staining of 3T3-L1 cells in the presence of Emdogain® and TGF-β.

### Statistical Analysis

Experiments were repeated at least twice and data are reported as the mean and standard deviation. ANOVA and hoc testing were used for analysis. Statistical significance was established at P<0.05.

## Results

### Emdogain® Inhibits 3T3-L1 Adipocyte Differentiation

**Figure 1:**
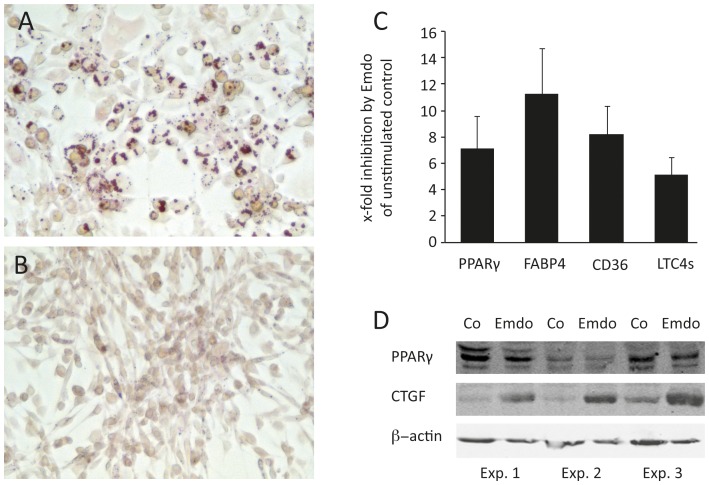
Emdogain® inhibits 3T3-L1 adipocyte differentiation. The 3T3-L1 murine preadipocytes were incubated with growth medium containing 1-methyl-3-isobutyl-xanthine dexamethasone and insulin for 5 days in the presence of (A) propylene glycol alginate or (B) Emdogain® at dilutions equivalent to 100 µg/ml and stained for lipid droplets. (C) Emdogain® reduced the expression of lipid regulated genes and (D) also the signal of PPARγ by Western blot analysis. Emdogain®, in contrast, increased the signal of CTGF.

To investigate the impact of Emdogain® on adipocyte differentiation, we determined the accumulation of intracellular lipids. The 3T3-L1 cells accumulated lipid droplets within 5 days among treatment with the adipogenic medium. Treatment of 3T3-L1 cells with Emdogain® almost completely suppressed the formation of lipid droplets (Figure 1A,B). Based on the TaqMan® array, Emdogain® considerably (<4-fold) decreased the mRNA level of PPARγ, FABP4, CD36, and LTC4s (Table 1). The data were confirmed by the traditional RT-PCR approach (Figure 1C). The decrease of PPARγ by Emdogain® was also confirmed at the protein level (Figure 1D).

**Table 1:** The 3T3-L1 murine preadipocytes were incubated with growth medium containing 1-methyl-3-isobutyl-xanthine dexamethasone and insulin for 5 days in the presence of (A) propylene glycol alginate or (B) Emdogain® at dilutions equivalent to 100 µg/ml and subjected to a TaqMan® Array for Mouse Lipid Regulated Genes.

Gene ID	fold upregulated	Gene ID	fold down regulated
Alox15	18	Soat2	2.4
Cd36	4.6	Insig1	2.5
Ltc4s	4.4	Fads3	2.5
Fabp4	4.2	Ptgs2	3.3
Pparg	3.8	Acat1	3.7
Nr1h3	3.5	Il6	4.7
Srebf1	3		
Fabp5	2.8		

The table indicates the genes at least 2-fold up-, or down regulated.

Emdogain® and TGF-β also increase CTGF in 3T3-L1 cells (Figure 1D). The TaqMan® array further revealed a 20-fold reduction of 15-lipogygenase (Alox15), which is a key enzyme converting arachidonic acid to the biologically active 15(S)-HETE [29]. However, this finding could not be confirmed by the traditional RT-PCR approach. Together, the data show that Emdogain® inhibits adipocyte differentiation of 3T3-L1 cells in vitro.

### Rosiglitazone and Indomethacin Failed to Rescue the Adipocyte Differentiation of Emdogain®-treated Cells

**Figure 2:**
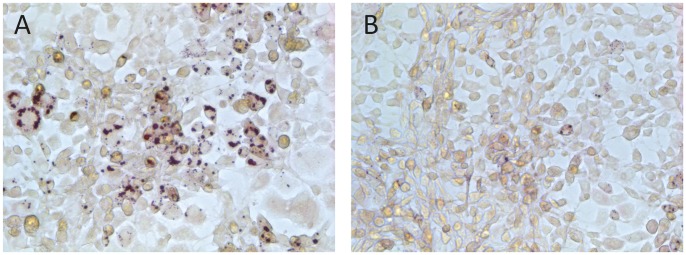
Rosiglitazone and indomethacin failed to rescue the adipocyte differentiation of Emdogain®-treated cells. The 3T3-L1 murine preadipocytes were incubated with growth medium containing indomethacin and rosiglitazone together with the basal adipogenesis-inducing medium for 5 days in the presence of (A) propylene glycol alginate or (B) Emdogain® at dilutions equivalent to 100 mg/ml and stained for lipid droplets.

To understand if the inhibitory effect of Emdogain® can be overcome by manipulation of PPARγ, rescue experiments with the supplementation of indomethacin, known to increase PPARγ expression [29], and rosiglitazone, an agonist for PPARγ [30], were performed. As shown in Figure 2, indomethacin and rosiglitazone, either alone or in combination, failed to provoke the formation of lipid droplets in 3T3-L1 cells treated with Emdogain®. Overall, the data show that targeting PPARγ by rosiglitazone and indomethacin cannot overcome the inhibitory effect of Emdogain® on adipogenic differentiation.

### SB-431542, an Inhibitor of TGF-β RI Kinase Activity, Reversed the Inhibitory Effects of Emdogain®

**Figure 3:**
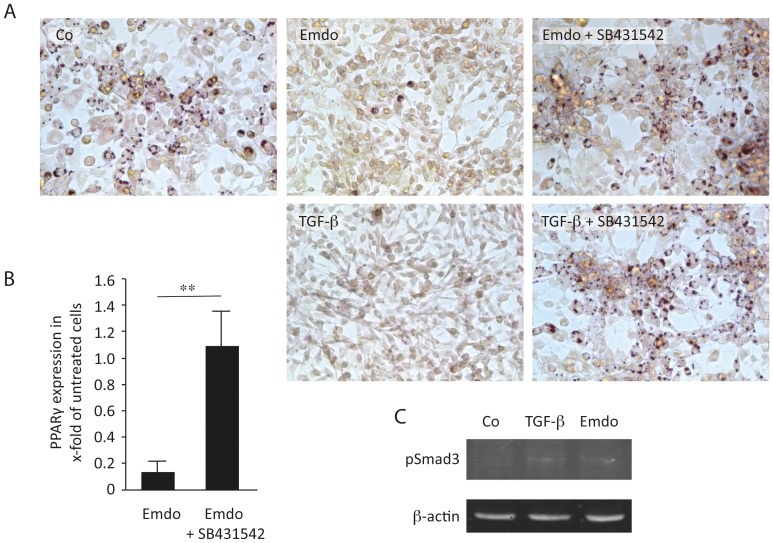
SB-431542, an inhibitor of TGF-β RI kinase activity, reversed the inhibitory effects of Emdogain®. The 3T3-L1 murine preadipocytes were incubated with basal adipogenesis-inducing medium containing rosiglitazone and indomethacin for 5 days in the presence of Emdogain® (100 µg/ml) or TGF-β (10 ng/ml) and a selective inhibitor of the TGF-β type I receptor SB-431542 (10 nM). (A) Lipid staining; (B) SB-431542 overcomes the blocking effect of Emdogain® on PPARγ expression; (C) Emdogain® and TGF-β increase phosphorylation of Smad3 in 3T3-L1 cells. **P<0.01%.

To dissect the mechanism by which Emdogain® decreases adipogenic differentiation of 3T3-L1 cells, we used the potent and selective inhibitor of the TGF-β type I receptor activin receptor-like kinase ALK5 [31]. SB-431542 allowed the formation of lipid droplets in the presence of Emdogain® (Figure 3A). In line with these findings, SB-431542 reversed the inhibitory effects of Emdogain® on the mRNA level of PPARγ in 3T3-L1 cells (Figure 3B). Also in primary murine fat-derived mesenchymal cells, SB-431542 reversed the inhibitory effects of Emdogain® and TGF-β on the expression of adipogenic genes (data not shown). Moreover, Emdogain® increased the mRNA levels of TGF-β by approximately 2-fold (data not shown). Immunoassay showed positive signals equivalent to approximately 100 ng/ml TGF- β1 in the commercial available Emdogain stock. In line with these data, Emdogain® and TGF-β increase phosphorylation of Smad3 in 3T3-L1 cells (Figure 3C). These results suggest that TGF-β signaling mediates the inhibitory effect of Emdogain® on adipogenic differentiation.

### Emdogain®-induced TGF-beta Signaling Cascade Increases CTGF Expression

**Figure 4:**
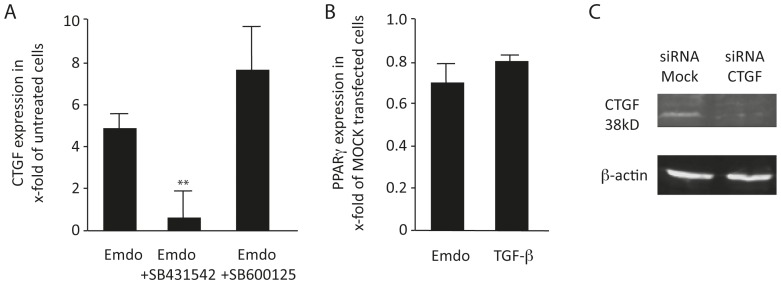
The Emdogain®-induced TGF-β signaling cascade increases CTGF expression. (A) The 3T3-L1 murine preadipocytes were exposed to Emdogain® (100 µg/ml) either with or without SB431542 or SB600125 for 18 hours in serum-free medium and the expression CTGF determined. (B) 3T3-L1 cells were transfected with siRNA CTGF and the respective MOCK siRNA before being stimulated with Emdogain® or TGF-β. (C) Inhibition of 38 kDa CTGF based on Western blot analysis.

To further support the involvement of the TGF-β signaling cascade, we examined the effect of Emdogain® on the expression of CTGF, which is highly regulated by TGF-β, and can inhibit adipogenesis [16], [23]. Emdogain® substantially increased the expression of CTGF in 3T3-L1 cells. Consistent with the central role of the TGF-β signaling cascade, the presence of SB431542 but not SB600125 failed to substantially affect CTGF expression in 3T3-L1 cells (Figure 4A). Moreover, 3T3-L1 cells transfected with siRNA CTGF showed higher expression levels of PPARγ when exposed to Emdogain® and TGF-β. Moreover, when transfected cells were exposed to Emdogain, a decrease of 38 kDa CTGF was observed by Western blot analysis (Figure 4C). However, siRNA CTGF could not counteract the suppression of lipid formation by Emdogain® and TGF-β (data not shown). These results indicate that the inhibition of adipogenic differentiation by Emdogain® may involve an increased expression of CTGF, at least in part by activation of TGF-β type I receptor activity.

### Emdogain® after Heating at 96°C Maintains the Ability to Activate TGF-beta Signaling

**Figure 5:**
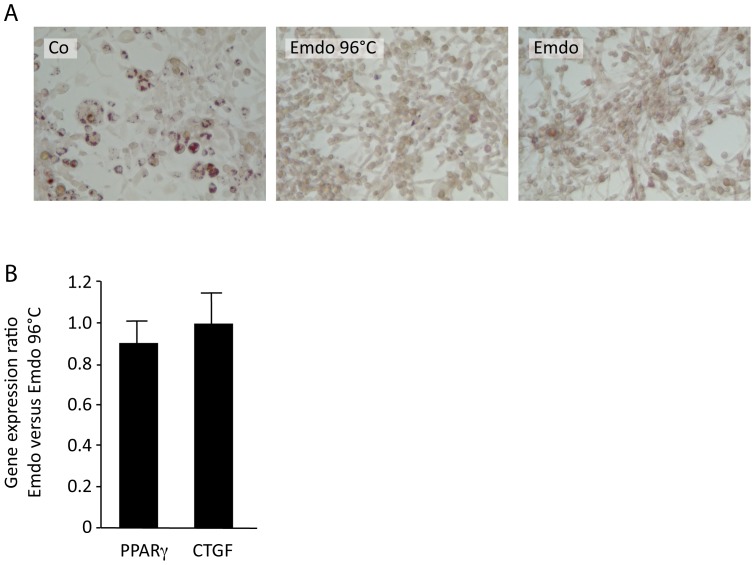
Emdogain® after heating at 96°C maintains the ability to activate TGF-β signaling. (A) The 3T3-L1 murine preadipocytes were exposed to Emdogain® (100 µg/ml) or Emdogain® previously heated up to 96°C within a basal adipogenesis-inducing medium containing rosiglitazone and indomethacin for 5 days and lipid staining was performed. (B) 3T3-L1 cells were also exposed to normal or heat-treated Emdogain® for 18 hours in serum-free medium before the expression of PPARγ and CTGF were determined.

Recent studies have shown that similar to TGF-β [28], also heat-treatment of Emdogain® maintains a biological activity in vitro [32]. We therefore determined whether heating up to 96°C can alter the anti-adipogenic activity of Emdogain®. As shown in Figure 5A, heat-treated Emdogain® almost abolished the formation of lipid droplets in 3T3-L1 cells, again being reversed by SB431542. Moreover, heat-treated Emdogain® similarly changed expression of PPARγ and CTGF compared to unheated Emdogain® (Figure 5B). SB431542 also reversed the effects of heat-treated Emdogain® on PPARγ and CTGF expression (data not shown). These findings further support the role of Emdogain® activating TGF-β RI kinase activity.

### 3T3-L1 Cells Retain their Adipogenic Potential after Transient Exposure to Emdogain®

**Figure 6:**
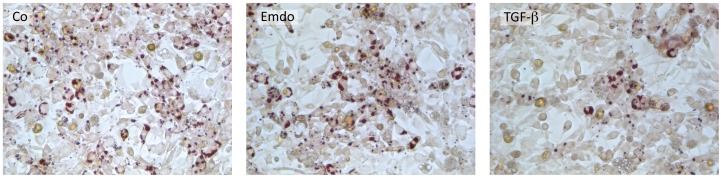
3T3-L1 cells retain their adipogenic potential after transient exposure to Emdogain®. The 3T3-L1 murine preadipocytes were incubated for one day with growth medium containing Emdogain® (100 µg/ml) or TGF-β (10 ng/ml). Then the medium was replaced by basal adipogenesis-inducing medium containing rosiglitazone and indomethacin for 5 days and lipid staining was performed.

Although adipogenic differentiation of 3T3-L1 cells is suppressed in the presence of Emdogain®, it is possible that the cells regain the capacity to form adipocytes once Emdogain® has been removed. Consistent with this idea, 3T3-L1 cells that were exposed to Emdogain® or TGF-β for 24 and 72 hours maintain their capacity to form lipid droplets when cultivated in the adipogenic medium. These results demonstrate that the inhibition of adipogenic differentiation of 3T3-L1 cells by Emdogain® is transient (Figure 6).

## Discussion

Initial studies showed that Emdogain® can suppress in vitro adipogenic differentiation of C2C12 cells [8], periodontal ligament fibroblasts [6] and palate fibroblasts (manuscript in preparation). TGF-β mediating at least part of the cellular responses to Emdogain® [11]–[14] is a potent inhibitor of adipocyte differentiation in vitro [15], [16]. The existing data raised the possibility that the inhibitory effects of Emdogain® on adipogenic differentiation involves TGF-β. The in vitro evidence presented here demonstrates that blocking TGF-β RI kinase activity does indeed overcome the inhibitory effect of Emdogain® on adipogenic differentiation of 3T3-L1 cells. Our observation that the amount of CTGF mRNA, a TGF-β regulated inhibitor of adipogenesis [16], [23], was also affected by Emdogain®, further supports the central role of TGF-β RI kinase activity in the suppression of adipogenic differentiation.

The present work is consistent with reports showing that Emdogain® strongly increases expression of CTGF in osteogenic cells [14] and data from microarray screening in periodontal ligament fibroblasts [33], [34], and epithelial cells [35]. It is, however, important to note that in the present study, siRNA CTGF only partially reduced the expression levels of PPARγ when exposed to Emdogain® and TGF-β, and that siRNA CTGF could not counteract the suppression of lipid formation by Emdogain® and TGF-β our observation basically supports the involvement of the TGF-β – CTGF autocrine pathway to mediate the inhibition of Emdogain® on adipogenesis, the definitive prove remains open. Thus, additional work will be required to determine whether complete blocking of CTGF can reverse the inhibitory effect of Emdogain® on adipogenic differentiation of mesenchymal progenitor cells in vitro.

The question arises about the source of TGF-β that mediates the inhibition of adipogenesis in vitro. In line with our findings that Emdogain® heated to 96°C maintains the respective biological activity supporting observations that TGF-β is stable under the same conditions [28]. Likewise, also heat-treatment of Emdogain® maintains a biological activity in vitro in other studies [32]. In support of this finding, the immunoassay detected >100 ng TGF-β1 in the Emdogain stock, resulting in at least 1 ng TGF-β1 in the working solution. Moreover, Emdogain caused the rapid phosphorylation of Smad3, which represents a mainly TGF-β signaling pathway. It is, then, possible that the inhibition of adipogenesis in our studies was the result of an intrinsic TGF-β activity of Emdogain, similar to other reports [10]. However, in vitro studies have suggested that Emdogain® can increase the expression of TGF-β is various cell types [10]. These previous findings are consistent with our data that TGF-β expression in 3T3-L1 cells is increased in response to Emdogain. Thus, there are two possible sources of TGF-β that can contribute to the overall inhibition of adipogenesis of mesenchymal cell in the present report.

Mesenchymal progenitors represent a population of cells within periodontal tissues and connective tissue grafts [5]–[7]. Since histological and clinical data have indicated that the combination of Emdogain® with palatal CTG may promote periodontal wound healing/regeneration in recession defects [1], [36], [37] the question arises, if Emdogain® affects adipogenic differentiation of mesenchymal progenitors in vivo. Moreover, adipose tissue-derived mesenchymal cells are used in tissue engineering [38], [39] and the transplanted cells usually should not regain their adipogenic phenotype. Hence, Emdogain® might serve as a potential carrier suppressing the formation of fat cells. However, our findings that the inhibition of adipogenesis by Emdogain® is only transient indicate the complexity of the interpretation of our findings. The setting of 24 hours cell incubation was chosen because also in vivo, Emdogain® remains in place for a limited time period. Until now, ectopic models with bone substitutes and mesenchymal cells, not isolated from adipose tissue, either alone or together with Emdogain®, have not reported on the differentiation of the cells toward adipocytes [40], [41]. Albeit the present study and in vitro work from others [6], [8] argue for a strong anti-adipogenic effect of Emdogain®, the clinical relevance remains a matter of speculation.

JNK signaling can mediate Emdogain® effects in vitro [21], and mitogen-activated protein kinase signaling is involved in TGF-β-mediated inhibition of adipogenesis [20]. Our studies showed that blocking of JNK signaling failed to modulate the increased expression CTGF upon incubation of the cells with Emdogain®. Based on this observation we propose that the classical Smad signaling plays the key role in mediating the effect of Emdogain® in the present study. Future studies are required to reveal the possible activation of the Smad signaling in addition to siRNA blocking of Smad 2 and Smad 3. Also the link of CTGF to signal via fibroblast growth factor (FGF) receptors-2 is relevant as bFGF is a antagonist for adipogenic differentiation of 3T3-L1 cells [42]. Future research perspectives might also reveal which size fraction of Emdogain® accounts true for the inhibition of adipogenesis.

In summary, we propose that the decreased adipogenesis by Emdogain® is caused by activation of TGF-β RI kinase in 3T3-L1 cells and is linked with a strong induction of CTGF expression. The present in vitro findings may serve as a primer for preclinical studies aiming to control adipogenic differentiation particularily in the field of regenerative dentistry.
